# Selfish chromosomal drive shapes recent centromeric histone evolution in monkeyflowers

**DOI:** 10.1371/journal.pgen.1009418

**Published:** 2021-04-22

**Authors:** Findley R. Finseth, Thomas C. Nelson, Lila Fishman

**Affiliations:** 1 Division of Biological Sciences, University of Montana, Missoula Montana, United States of America; 2 Keck Science Department, Claremont-McKenna, Scripps, and Pitzer Colleges, Claremont California, United States of America; Fred Hutchinson Cancer Research Center, UNITED STATES

## Abstract

Centromeres are essential mediators of chromosomal segregation, but both centromeric DNA sequences and associated kinetochore proteins are paradoxically diverse across species. The selfish centromere model explains rapid evolution by both components via an arms-race scenario: centromeric DNA variants drive by distorting chromosomal transmission in female meiosis and attendant fitness costs select on interacting proteins to restore Mendelian inheritance. Although it is clear than centromeres can drive and that drive often carries costs, female meiotic drive has not been directly linked to selection on kinetochore proteins in any natural system. Here, we test the selfish model of centromere evolution in a yellow monkeyflower (*Mimulus guttatus*) population polymorphic for a costly driving centromere (*D*). We show that the *D* haplotype is structurally and genetically distinct and swept to a high stable frequency within the past 1500 years. We use quantitative genetic mapping to demonstrate that context-dependence in the strength of drive (from near-100% *D* transmission in interspecific hybrids to near-Mendelian in within-population crosses) primarily reflects variable vulnerability of the non-driving competitor chromosomes, but also map an unlinked modifier of drive coincident with kinetochore protein Centromere-specific Histone 3 A (CenH3A). Finally, CenH3A exhibits a recent (<1000 years) selective sweep in our focal population, implicating local interactions with *D* in ongoing adaptive evolution of this kinetochore protein. Together, our results demonstrate an active co-evolutionary arms race between DNA and protein components of the meiotic machinery in *Mimulus*, with important consequences for individual fitness and molecular divergence.

## Author summary

Centromeres must mediate faithful chromosomal transmission during cell division and sexual reproduction, but both the DNA and protein components of centromeres diverge rapidly across species. The selfish centromere model argues that this paradoxical diversity results from a genetic conflict between centromeric DNA variants driving through female meiosis to gain over-transmission and kinetochore proteins co-evolving to re-establish Mendelian segregation. We use whole genome sequencing and genetic crossing experiments to demonstrate active evolutionary interactions between a selfish centromere and a key kinetochore protein (CenH3A) in the wildflower *Mimulus guttatus*. We show that both inter-specific and intra-population differences in CenH3A affect centromeric drive in hybrids, and that adaptive evolution of CenH3A has followed the recent and costly spread of the driver in a wild population. This work provides novel empirical support for the proposed antagonistic co-evolution of the DNA and protein components of centromeres, with important consequences for understanding cellular function, individual fitness, and species divergence.

## Introduction

Centromeres, which mediate the conserved and essential processes of chromosomal segregation during eukaryotic mitosis and meiosis, are paradoxically diverse. Centromeric DNA arrays are highly variable in sequence, size, and position, and the protein that epigenetically marks the site of kinetochore assembly, Centromere-specific Histone 3 (CenH3; known as CENP-A in humans), commonly evolves under diversifying selection [1–3]. The selfish centromere hypothesis [2,4] resolves this paradox by arguing: a) asymmetric female meiosis creates an arena for selection among homologous centromeres for inclusion in the single egg cell, b) female meiotic drive is costly to individuals and c) costs of drive promote suppressive coevolution by CenH3 and other key kinetochore proteins. This model of genetic conflict between the DNA and protein components of centromeres has profound implications for the maintenance of individual fitness variation, the divergence of species, and the evolution of genomes and cellular processes [reviewed in 5–7]. Furthermore, understanding centromere function and evolution directly impact human endeavors from cancer therapies [8] to crop improvement [9]. However, despite recent advances in understanding the molecular biology [10,11] and evolutionary dynamics [12] of centromeric drive, evidence for the posited evolutionary arms race between centromere DNA and kinetochore proteins remains largely circumstantial. Here, we directly test the key final step of the centromere drive hypothesis in a flowering plant with an active (and costly) driving centromere.

In the yellow monkeyflower, *Mimulus guttatus* (Phrymaceae), the *D* allele on Linkage Group/Chromosome 11 (LG11) drives through female meiosis against both conspecific (*M*. *guttatus D*^*-*^ allele; *D*: *D*^*-*^ female transmission ratio = 58:42) and heterospecific (M. *nasutus d* allele; *D*:*d* ratio > 98:2) alternative alleles [13,14]. *D* is genetically and cytogenetically associated with dramatically expanded arrays of the *M*. *guttatus* centromere-specific DNA repeat Cent728 [14,15]. In addition, near-perfect transmission in heterospecific F_1_ hybrids, which is only possible via centromeric drive in Meiosis I [16], strongly suggests that the drive locus (hereafter Meiotic Drive Locus 11; MDL11) acts as the centromere of LG11 in intraspecific and interspecific heterozygotes. The driving *D* is prevented from fixation and maintained at 35–45% in our focal annual Iron Mountain (IM) *M*. *guttatus* population (Oregon Cascades, USA) by homozygous costs to both male fitness (*DD* pollen viability cost = 20%) and female fertility (*DD* seedset cost = 14–23%) [12,14]. Recent genome-wide association mapping of flowering traits in the field found little or no effect of *D* on other fitness components [17], confirming that its evolutionary dynamics primarily reflect a balance between selfish female meiotic drive and fertility costs. Because a costly driver at a polymorphic equilibrium generates selection for unlinked suppressor loci [18], this population provides the ideal opportunity to assess the consequences of centromeric drive for selection on linked and unlinked genes.

## Results/Discussion

**Fig 1:**
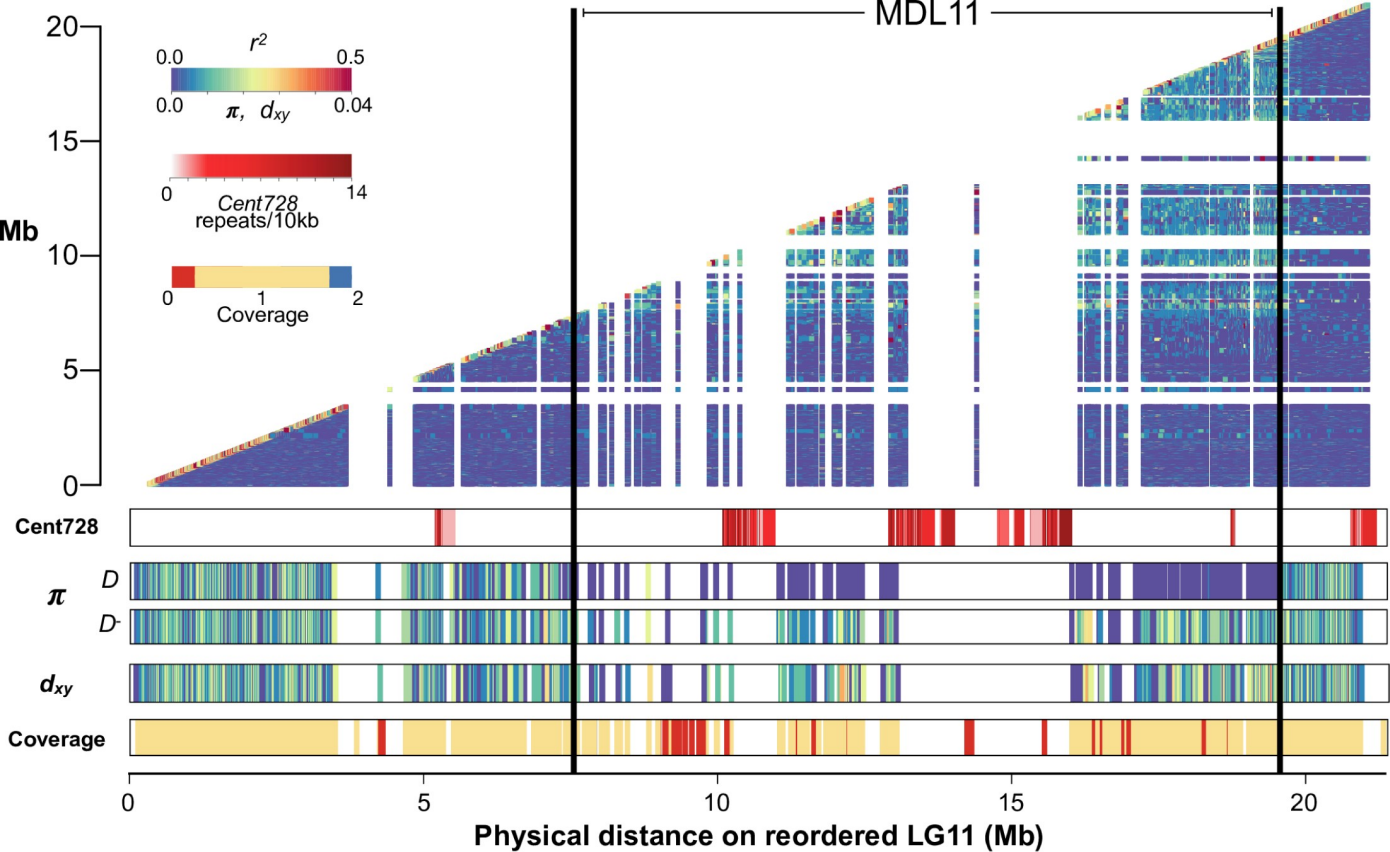
The *Mimulus guttatus* centromeric driver (*D*) is an extended low-recombination haplotype with distinct sequence content. Top panel: Suppressed recombination between driving *D* and non-driving *D*^*-*^ haplotypes causes elevated linkage disequilibrium (*r*^*2*^) across Meiotic Drive Locus 11 (MDL11) in the Iron Mountain (IM) population of *M*. *guttatus* (heatmap of *r*^*2*^ plotted by megabase position on x- and y-axes; N = 34 inbred lines). Lower panels, from top to bottom: the chromosome-wide density of putatively centromeric Cent728 repeats in the *D* reference genome; nucleotide diversity (π) per gene for *D* (n = 14) and *D*^*-*^ lines (n = 20); divergence (*d*_*x*,*y*._) per gene between *D* and *D*^*-*^ lines; and the ratio of exon coverage in *D*^*-*^ lines vs. D lines when aligned to the *D* reference genome (values near zero suggest deletion in *D*^*-*^ vs. *D* haplotype, whereas values near 2 suggest duplication).

Comparative linkage mapping demonstrates local suppression of recombination in F_1_ hybrids of the IM62 *M*. *guttatus* reference line (*D*) with *D*^*-*^ and *d* lines [19], suggesting that the *Cent728* expansions associated with *D* are embedded in a chromosomal rearrangement (likely an inversion) that reduces chromosomal pairing or crossing over between alternative haplotypes. Because the *M*. *guttatus* reference genome sequence was assembled into chromosome-scale scaffolds using a locally non-informative *D* x *D*^*-*^ linkage map, we generated a corrected LG11 genome order based on a collinear *D*^*-*^ x *D*^*-*^ map (S1 Table) [19]. Using this collinear (but likely inverted relative to *D* chromosomes) order, IM inbred lines exhibit a contiguous block of elevated linkage disequilibrium (LD) across the region of LG11 corresponding to the driving *D* haplotype (MDL11: Figs 1 and S1 and S2 Table). Although containing more than half of Chromosome 11 DNA sequence, this >12 Mb block almost certainly underestimates the true physical extent of MDL11. In the *D* reference genome, this region contains extensive arrays of Cent728 repeats (Fig 1), but repetitive centromeric and peri-centromeric DNA are likely under-represented in the assembled and mapped genome scaffolds. Although we do not yet know which sequences in each MDL11 haplotype bind centromeric histones in different genetic contexts, the entire region (including both Cent728 arrays and genes; Fig 1) segregates with functional centromere in the *DD*^*-*^ and *Dd* heterozygotes where drive occurs.

As predicted by population genetic models [12] and previously inferred from a handful of marker sequences [14], the sweeping away of genetic variation across *D* demonstrates its rapid and recent spread to intermediate frequency despite substantial individual fitness costs. Across MDL11, *D* lines are essentially invariant, whereas *D*^*-*^ lines are highly variable and both sets of lines exhibit high diversity in flanking regions (Table 1 and Figs 1 and S1). To estimate the age of the recently swept *D* haplotype within the IM population, we counted single nucleotide variants (SNVs) in coding sequence across the region in 13 *D* lines (S3 Table). In ~256 kb of unambiguously *D* coding sequence we identified 9 single nucleotide variants (SNVs) present in one or more lines. Using mutation rates = 0.2–1.5 x 10^−8^, following [20], this accumulation of variation corresponds to 200–1497 years (*M*. *guttatus* generations) since the sweep with simple population genetic equations [21]. Forward simulations with similar parameters find a mean time to common *D* ancestor of 999 years (S2 Fig).

**Table 1:** Nucleotide diversity across LG11 in the IM *Mimulus guttatus* population.

Lines^a^	Region^b^	Mean π (SE)^c^	Mean *d*_*x*,*y*_ (SE)	95% CI^d^	N_genes_^e^
*D*	MDL11	0.0002 (0.00009)	--	(0.00007–0.0004)	219
*D*^*-*^	MDL11	0.0097 (0.0004)	--	(0.0089–0.0104)	219
*D*	Flanking	0.0096 (0.0002)	--	(0.0092–0.0100)	855
*D*	Flanking	0.0100 (0.0002)	--	(0.0096–0.0103)	855
*D* vs *D*	MDL11	--	0.0110 (0.0005)	(0.0102–0.0120)	231
*D* vs *D*	Flanking	--	0.0098 (0.0002)	(0.0094–0.101)	867

a 14 IM lines with D haplotype, 20 IM lines with D^-^ haplotype

b MDL11 = region of LG11 spanning driving D haplotype; Flanking = LG11 outside of MDL11

c Nei’s diversity per gene per site [22]

d Confidence intervals (CI) generated by resampling the mean without assuming normality (N = 1000)

e Number of genes without missing data

Given the distinctiveness of the *D* haplotype, it is worth considering whether it arose by local mutation, gene flow from another population, or introgression from another species. The *D* haplotype also occurs in at least one other intensively sampled population from the Oregon Cascades [23], suggesting that it may not have arisen by mutation within our focal population. However, both divergence estimates and coalescent models suggest that haplotype associated with drive is unusually extended and common, but not unusual in sequence or origin. Divergence (genic *d*_*x*,*y*_) between *D* and *D*^-^ lines is only marginally higher in MDL11 vs. flanking regions (0.011 vs. 0.0098; Table 1 and Fig 1). Further, while trans-specific introgression of other loci has been observed at Iron Mountain [24], it is unlikely to be an initiator of drive in *M*. *guttatus*. In pairwise coalescent analyses with samples from outside the IM population, the *D* and *D*^*-*^ haplotypes exhibit similar inferred demographic histories, both inside and outside MDL11 (S3 Fig). Further, consistent with no elevation of *d*_*x*,*y*_ across MDL11 (Table 1), there is no evidence of unusually deep coalescence between the sampled *D* and *D*^*-*^ haplotypes. Together, these results suggest that the driving *D* haplotype arose by structural and sequence mutation within the Northern clade of *M*. *guttatus* rather than from long-distance migration or interspecific introgression.

Given that the MDL11 region includes at least 387 protein-coding genes (Fig 1 and S4 Table), it is possible that linked genes enhance female meiotic drive and/or contribute to the substantial fitness costs of *D* homozygosity. Male meiotic drive factors, such as *Segregation Distorter* in fruit flies, are often associated with rearrangements that genetically link sperm-killing alleles with responder or enhancer genes [25]. Female meiotic drive, on the other hand, involves physical competition between structurally divergent chromosomes and thus does not require differences in gene sequence or expression. However, linked genic enhancers are predicted to accumulate whenever LD is high around any selfish element [18]. Furthermore, female meiotic drive by a neocentromeric driver in maize requires both a physical knob of heterochromatic satellite DNA and a cluster of kinesin genes, which are locked together within an inversion [26]. To assess the opportunity for collusion between driving *Cent728* arrays and linked genes, we surveyed MDL11 for genes with potential meiotic functions (S4 Table). Candidates include the sole *M*. *guttatus* homologue of Nuclear Autoantigenic Sperm Protein (NASP)/Sim3, which was recently identified as the chaperone of plant centromeric histones [27]. In addition, a > 800kb region (45 genes: Migut.K01214-Migut.K1259) present in *D* but exhibiting near-zero sequence coverage in all *D*^*-*^ lines (Fig 1 and S4 Table) contains a homologue of Arginine-Rich Cyclin RCY1, a component of the male-meiosis-essential Cyclin L/CDKG1 complex [28]. Thus, gene content differences between *D* and non-*D* haplotypes may also contribute to either drive or its reproductive costs. However, because all diagnostic *D* variants are equally associated with meiotic drive within the IM population and in hybrids, we cannot genetically uncouple these potential genic modifiers from the *Cent728* arrays. In the future, genetic editing of target sequences in *Mimulus* may make direct study of their drive-relevant functions possible.

Centromeric drive sets up a conflict of interest between the driver and genes genome-wide that bear its costs, with components of the kinetochore machinery particularly likely evolutionary interactors. In *M*. *guttatus*, the striking difference in the strength of drive between heterospecific and conspecific hybrids allows quantitative genetic investigation of this process over long time scales, while the costly drive polymorphism within IM can illuminate it from a population genetic perspective. Thus, we first ask whether unlinked suppressor loci contribute to the relative weakness of conspecific (*DD*^*-*^; 58:42) vs. heterospecific (*Dd;* 98:2) drive and then examine population genomic patterns of selection at a functional and positional candidate. These approaches are complementary: the quantitative genetic approach casts a broad net to assay accumulated differences between species but cannot distinguish driven co-evolution from other sources of epistasis in hybrids [29,30], while the population genomics is a single gene-scale snapshot of evolution in action.

**Fig 2:**
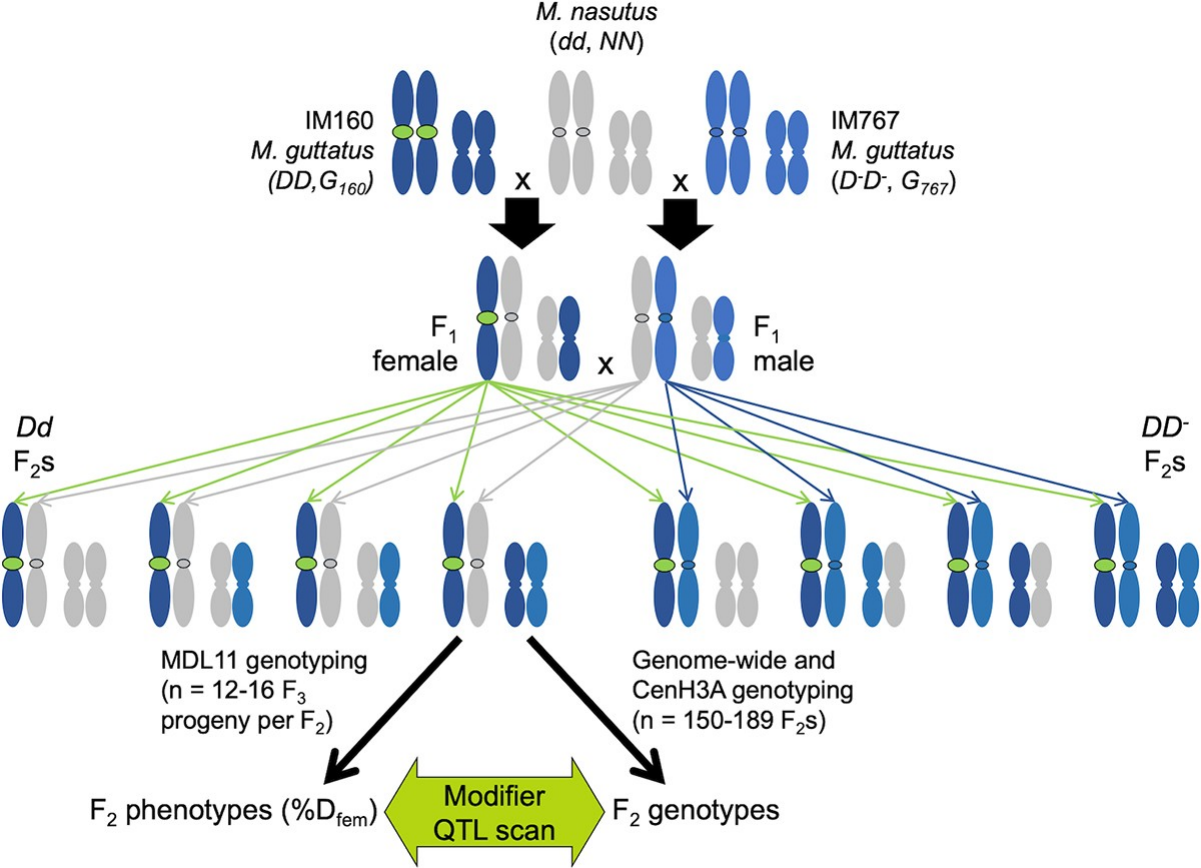
Crossing design for mapping unlinked modifiers of heterospecific (*Dd*) and conspecific (*DD*^*-*^) drive. Two pairs of chromosomes are shown: Chromosome 11 with the centromeric MDL11 locus outlined in black and a second pair representing the rest of the genome. *D* (IM160; dark blue and green) and *D*^*-*^ (IM767; pale blue) lines of *M*. *guttatus* were crossed to *M*. *nasutus* (grey) to generate heterospecific F_1_ hybrids. Intercrossing the F_1_s produced an F_2_ mapping population segregating only *DD*^*-*^ and *Dd* at MDL11 due to strong heterospecific drive through the female *Dd* parent: green arrows) and in Mendelian ratios elsewhere (blue and grey arrows). F_2_s were genotyped genome-wide (scored as NN, NG, GG) and at a marker that could distinguish the alternative *CenH3A* alleles donated by the IM160 and IM767 parents (G_160_ and G_767,_ respectively).

Because *M*. *nasutus* is a highly selfing species [20], centromeric drive and other forms of genetic conflict should have been relaxed since its split from *M*. *guttatus* [31]. Thus, centromeric or genic divergence within MDL11 alone (i.e. *M*. *guttatus D*^*-*^ vs. *M*. *nasutus d* as competitors with *D*) could govern the strength of transmission ratio distortion in *DD*^*-*^ vs *Dd* heterozygotes. However, *M*. *nasutus* alleles at unlinked loci may be particularly permissive to drive in F_1_ hybrids and *M*. *nasutus*-background nearly isogenic lines [13]. To evaluate these (non-exclusive) alternatives and map any unlinked modifier loci, we generated a three-parent interspecific F_2_ mapping population (Fig 2 and Methods). Briefly, we crossed a *Dd* F_1_ female parent (SF *M*. *nasutus* x IM160 *M*. *guttatus*) to a *D*^*-*^*d* F_1_ male parent (SF x IM767 *M*. *guttatus*), genotyped the F_2_ hybrids genome-wide using a reduced-representation sequencing method, and constructed a linkage map [19]. As expected, the *Dd* F_1_ female transmitted only *D* alleles to the next generation, and the F_2_ hybrids consisted entirely of *Dd* and *DD*^*-*^ individuals (n = 88 and 96, respectively). We used the frequency of *D* in selfed-F_3_ progeny of F_2_ hybrids (n = 12–16 genotyped per family, total N > 2400) to calculate the strength of female meiotic drive (%D_fem_, assuming male to be Mendelian). Averaged across genetic backgrounds in F_2_ siblings, *Dd* drive remained dramatically stronger than *DD*^*-*^ drive (mean %D_fem_ = 0.93 vs. 0.73; r^2^ = 0.26; n = 159). Thus, stronger drive against the *M*. *nasutus d* allele can primarily be ascribed to structural and/or genic divergence in the functionally centromeric MDL11 region. Thus, beyond the current dynamics of the *M*. *guttatus D* variant at MDL11, *M*. *nasutus* may have both generally “weak” centromeres and a cellular machinery that is particularly vulnerable to selfish elements.

**Fig 3:**
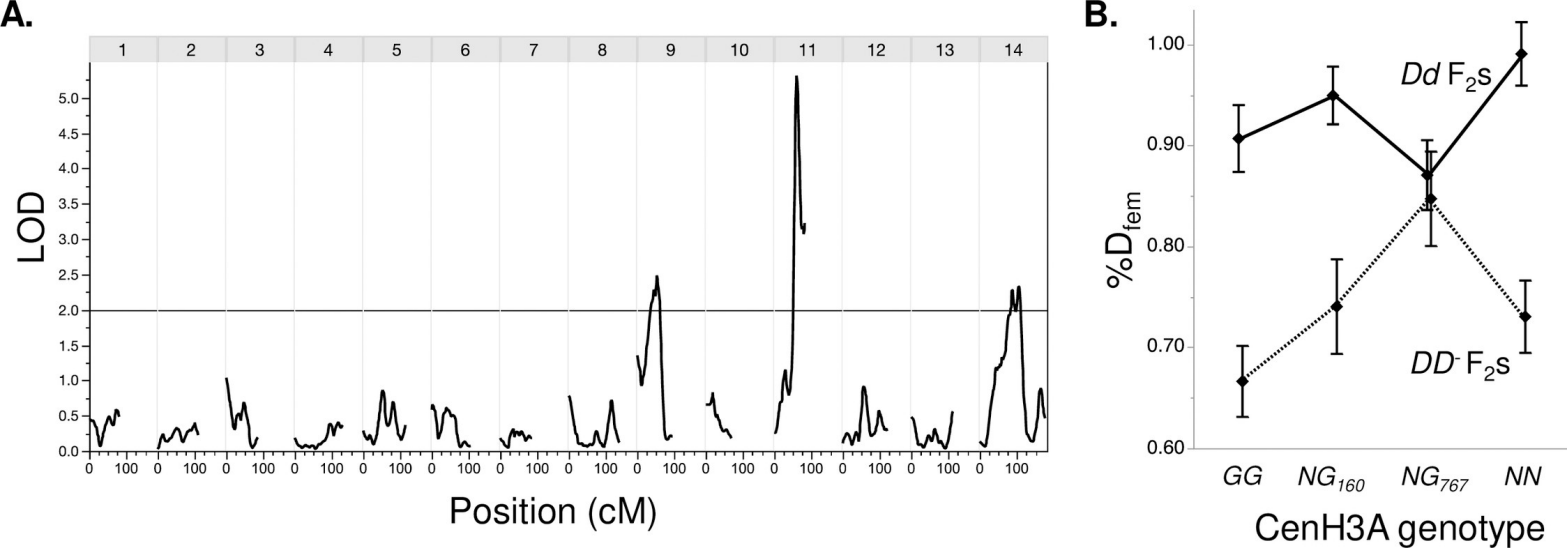
The strength of conspecific vs. heterospecific drive depends on MDL11 genotype, as well as unlinked modifiers. **(A)** A quantitative trait locus (QTL) scan of transmission ratio distortion in F_3_ progeny of F_2_ hybrids reveals unlinked modifier QTLs on Chromosome 9 and 14, in addition to the primary effect of MDL11 genotype. LOD score trace is smoothed, with a window size of four markers. **B** Genotype at CenH3A, which is centered under the Chromosome 14 modifier QTL, significantly influences *D* transmission in hybrids. Means ± 1 SE are shown for the eight F_2_ genotypic classes: *DD*^*-*^ and *Dd* at MDL11and *GG* (IM160/IM767 *M*. *guttatus*), *NG*_*160*_ (heterozygote with *M*. *guttatus* allele from IM160 parent), *NG*_*767*_ (heterozygote with *M*. *guttatus* allele from IM767 parent), and *NN* (M. *nasutus*) at CenH3A. Total n = 146.

Despite its primary effect, however, MDL11 genotype could not fully explain variation in the intensity of drive, suggesting that unlinked genetic modifiers also modulate drive in interspecific F_2_ hybrids. In our F_2_s, *Dd* drive (0.93) was reduced relative to the expectation from F_1_s and majority-*M*. *nasutus* isogenic lines (>0.98) [13], whereas *DD*^*-*^ drive was substantially elevated relative to our expectation from previous crosses within IM (mean %*D*_fem_ = 0.73 vs. 0.58) [14]. A scan for quantitative trait loci (QTLs) affecting *D* transmission detected weak unlinked modifiers on Chromosomes 9 and 14 (n = 130; LOD > 2.0; peak r^2^ = 0.09 for both; Fig 3A). The large confidence intervals (20–50 cM) around these minor QTLs span hundreds of genes, but the Chromosome 14 modifier QTL is notably centered over one of the two genes encoding CenH3 in *M*. *guttatus* and relatives (CenH3A) [3]. Because CenH3 proteins are the leading functional candidates for suppression of centromeric drive [2], we further characterized *Dd* and *DD*^*-*^ drive in all four CenH3A genotypes found in our F_2_ hybrids (*G*_*160*_*G*_*767*_ *M*. *guttatus* homozygote, *NG*_*160*_ and *NG*_*767*_ interspecific heterozygotes, and *NN M*. *nasutus* homozygotes as determined by diagnostic marker alleles; n = 146). We see a strong primary effect of MDL11 genotype (F_1,3_ = 47.20, P < 0.00001) and (marginally) the expected elevation of *D* transmission in *M*. *nasutus* vs. *M*. *guttatus* CenH3A homozygotes across both MDL11 genotypic classes (Least Squares Means comparison: *P* = 0.059; Fig 3B). In addition, CenH3A and MDL11 genotypes interacted non-additively (*F* = 3.91, interaction *P* < 0.01), with *DD*^*-*^ drive becoming as strong as *Dd* drive exclusively in *NG*_*767*_ heterozygotes (Fig 3B). Although the CenH3A allele from IM767 did not enhance conspecific drive when paired with a second *M*. *guttatus* allele, as in [32], this heterozygous effect may contribute to strong drive in interspecific F_1_ hybrids.

At this point, we cannot pinpoint the cause of any differential effects of CenH3A alleles on MDL11 drive; however, it is notable that the CenH3A allele from IM160 (which was chosen as a crossing parent only for its *D* genotype) happens to be substantially distinct in sequence from IM767, which is near-identical at CenH3A to the reference (*D*) line IM62 used in previous crosses (see Methods for more detail). The two *M*. *guttatus* lines differ by only a single nonsynonymous site in Exon 4 in the rapidly evolving N-terminal region. This site is one of many that differ between *M*. *nasutus* and IM767/IM62 (3), but is also one of very few CenH3A polymorphisms that appears to segregate at intermediate frequency in the IM population (9 of 33 IM lines carry the IM160/*M*. *nasutus* allele at the nonsynonymous Exon 4 site). While these CenH3A sequence differences provide opportunities for further functional investigation, it is not yet clear whether they (or other linked variants) influence drive in intraspecific contexts. Nonetheless, interactions between heterospecific CenH3A alleles intriguingly mirror the underdominant effects of CenH3 on meiosis in transgenic experiments transferring CenH3s among widely divergent plant species. In that work, *Arabidopsis* plants expressing homozygous maize CenH3 produce viable offspring when selfed, but engineered maize-*Arabidopsis* CenH3 heterozygotes exhibit zygotic mis-segregation, aneuploidy, and inviability [33], implying uniquely negative interactions between distinct versions of CenH3 during cell division. Similarly, our results suggest that sensitivity of meiosis to *within*-locus mismatch between heterospecific CenH3 alleles, on top of the posited role for *between*-locus mismatch between centromeric histones and centromeric DNA [2], may unmask meiotic drive in hybrids.

**Fig 4:**
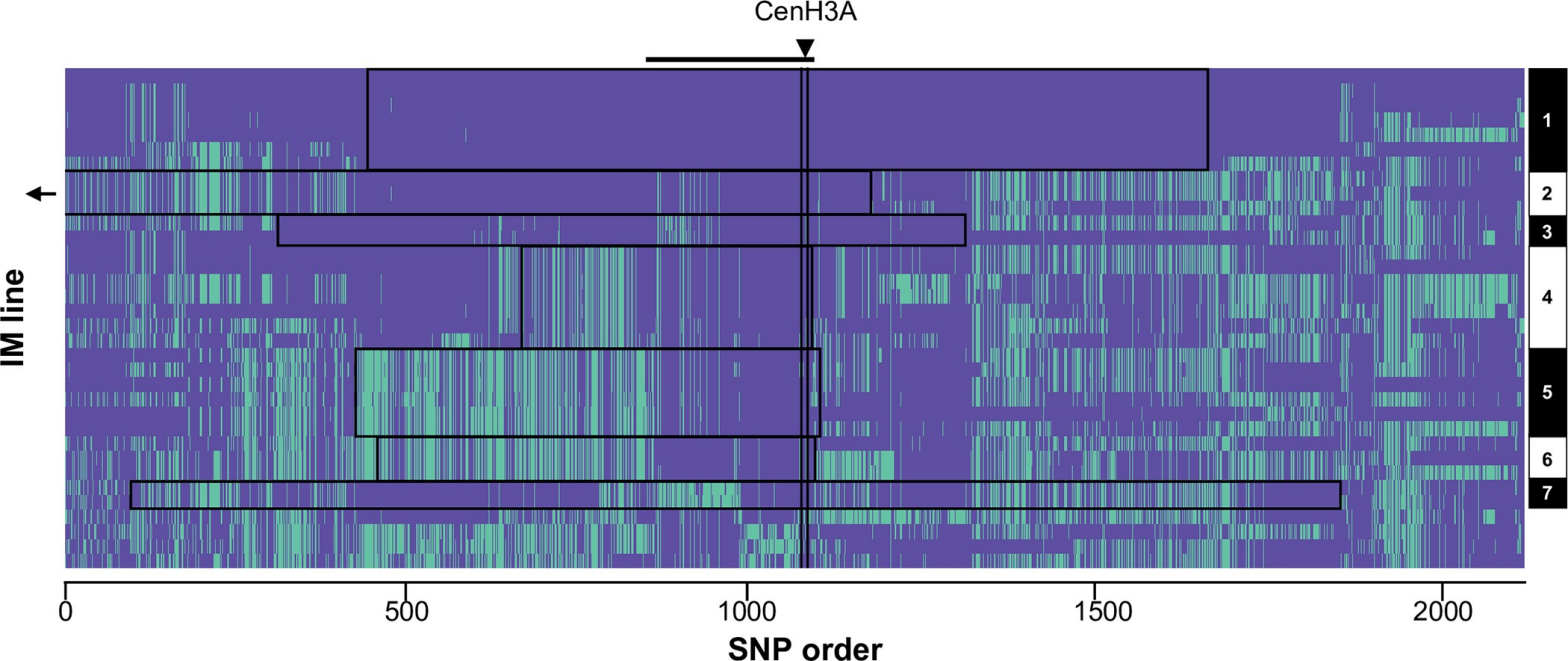
CenH3A exhibits a recent selective sweep, consistent with evolution in response to costly *D* spread. Exonic single nucleotide polymorphisms (SNPs) across a 496 kilobase (kb) region flanking CenH3A (13.5–14 Mb on Chromosome 14) are displayed for each of 34 lines from the Iron Mountain population of *M*. *guttatus*. The ~2000 SNPs are ordered by genomic position, but the x-axis is not scaled to physical or genetic distance. SNPs are coded according to whether they match (purple) or differ from (green) the haplotype of IM1054, which bears one of the most common CenH3A-flanking haplotypes. The arrowhead and horizontal line mark the location of CenH3A. The seven haplotypes (1–7) were assigned manually and are outlined in black boxes. For visual resolution around CenH3A, the longest haplotype (> 620kb) was truncated. Haplotype details are given in S6 Table.

While quantitative genetic modification of drive by linked and unlinked genes in *M*. *nasutus* x M. *guttatus* hybrids likely reflects evolution in both species, the spread of *D* (with its costs) specifically predicts signatures of recent selection on interacting loci within the Iron Mountain *M*. *guttatus* population. We examined the two centromeric histones, as they are primary functional candidates for antagonistic co-evolution with costly *D* chromosomes and CenH3A is also a candidate modifier in the mapping experiment. Strikingly, an 8-gene region (Migut.N01552-Migut.N01559) containing CenH3A (Migut.N01557) exhibits a near-complete selective sweep at IM (Fig 4 and S4 Fig), whereas CenH3B shows no signatures of local selection [3,24]. The CenH3A region is an outlier in within-population nucleotide diversity (mean π: 0.00232, *P* < 0.017) and has a significantly skewed site frequency spectrum (mean Tajima’s D: -0.838, P < 0.017, S4B Fig), but exhibits typical inter-population diversity (*P* > 0.05 in all comparisons; S5 Table). These signatures, along with elevated linkage disequilibrium (Fig 4), indicate a recent local selective sweep rather than widespread purifying selection.

To age the CenH3A selective sweep relative to that of *D*, we considered two scenarios. First, if the 23.9 kb core region shared by all swept haplotypes decayed from single whole-chromosome haplotype following the introduction of a novel mutation now near fixation, the selective sweep at IM occurred 627–4178 years ago, depending on the local recombination rate (Methods). However, strong haplotype structure extends across a substantially larger flanking region around CenH3A (Fig 4), suggesting that novel selection likely favored a standing variant found on multiple genetic backgrounds. Seven distinct long-range haplotypes of CenH3A were represented by two or more lines (Fig 4 and S6 Table), and the median and mean lengths of these haplotypes (164.3 kb and 221.1kb, respectively) support a more recent response to novel selection (ranges = 91–609 and 68–452 years ago, respectively, depending on the local recombination rate; see Methods). Of course, the history of selection on CenH3A may be more complex than either of these scenarios. CenH3 sequences routinely exhibit the recurrent positive selection detected by measures of long-term molecular evolution [3], and *D* may not be the only selfish centromere exerting selection in *M*. *guttatus*, or even at Iron Mountain. Nonetheless, the timescales estimated under either hard (new mutation) or soft (standing variation) sweep scenarios are consistent with the hypothesis that the recent (~1000 years) spread of *D* to intermediate frequency sparked selection on CenH3A variation.

**Fig 5:**
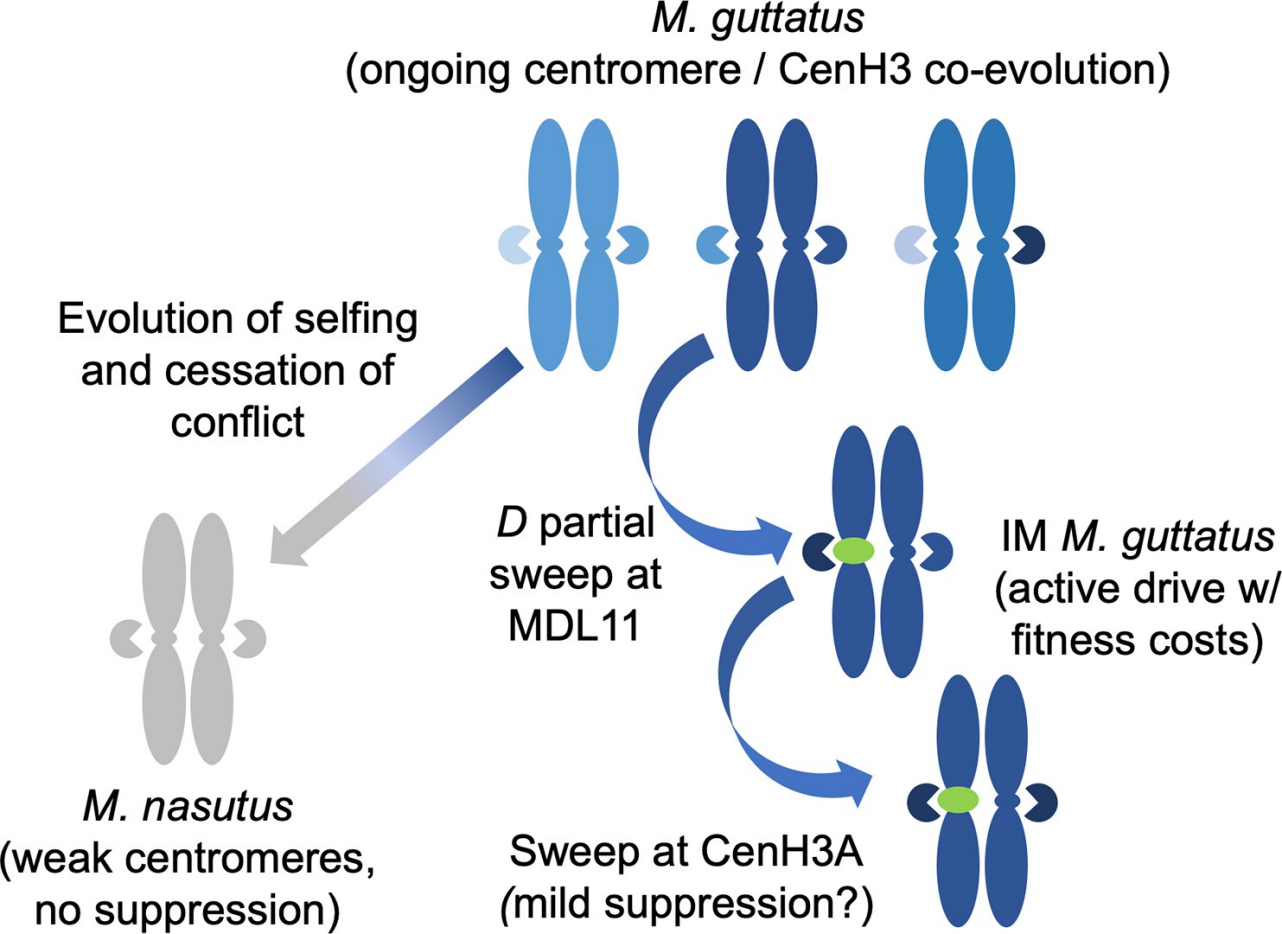
Hypothesized processes underlying differences between heterospecific (strong) and conspecific (weak) centromeric drive in yellow monkeyflowers, as well as population genetic signatures of recent *D*-CenH3A co-evolution in Iron Mountain (IM) *Mimulus guttatus*. Shades of blue represent *M*. *guttatus* standing diversity at centromeres (ovals on chromosomes), CenH3A (pie shapes), and other loci, green represents the driving *D* centromere (potentially facilitated by linked genes), and grey represents the relatively drive-permissive centromere and genetic background of selfer *M*. *nasutus*.

Overall, our results demonstrate that genic factors can modify the strength of centromeric drive in hybrids and that the recent spread of a selfish chromosome has plausibly driven local evolution of a key kinetochore protein in a wild plant (Fig 5). Thus, both quantitative and population genetic lines of evidence from *Mimulus* support influential models in which centromeres routinely drive through asymmetric female meiosis, with fitness consequences that select for compensation by other components of the segregation machinery. Over the long timescale of species divergence, likely exacerbated by the relaxation of both conflict and purifying selection on the meiotic machinery following the evolution of selfing, we see the development of extreme vulnerability to centromeric drive in *M*. *nasutus*. This occurs primarily at the drive locus itself, but *M*. *nasutus* homozygotes at CenH3A and one other locus are also relatively permissive of drive. Over the more recent timescale of a single *M*. *guttatus* population, CenH3A shows a recent selective sweep consistent with selection to respond to the costs of centromeric drive. We also note differences in how different *M*. *guttatus* CenH3A alleles interact with the meiotic drive locus in interspecific hybrids but cannot yet connect that variation to the evolutionary dynamics of drive and suppression within *M*. *guttatus*. Together with prior work on costs of drive, these results reveal all predicted steps of the original paradox-resolving centromeric drive model in action in a natural population and illustrate a key role for centromeric histones in modulating the selfishness of chromosomes.

Paradoxically, the aspects of the *Mimulus D* drive system that allow illumination of centromere-CenH3 coevolution in action may be atypical of the ubiquitous centromeric drive posited to drive divergence in centromeric repeats and proteins between species [2]. All three components of the centromere drive model occur simultaneously in our focal population of *Mimulus guttatus* only because *D* carries recessive costs that brake its spread and create a balanced polymorphism at intermediate frequency [12,34]. This equilibrium generates persistent recessive costs that provide time (and selection) for the rise and spread to fixation of even weak suppressor mutations [18]. Under most other cost regimes (e.g., heterozygous costs of drive *per se*), even an undetectably weak centromeric driver would sweep to fixation long before a suppressor could arise by mutation (or, if heterozygous costs outweighed drive, be deterministically lost from the population). Our finding that the CenH3A selective sweep at IM likely involving a standing variant found on multiple haplotypic backgrounds is consistent with this fundamental time constraint; the alternative of a novel beneficial variant sweeping would require that a fortuitously favorable suppressor-of-centromeric-drive mutation hit the miniscule sequence target of a few core meiotic proteins within the past 1000 years. Further investigation of population-level variation in CenH3 across species provides a potential avenue for linking within-population dynamics to species level centromere divergence.

Finally, the particularities of the *Mimulus* drive system, as well as its match to general predictions, re-iterate that the nature of costs is key to understanding the evolutionary dynamics of driving centromeres. If drive has no pleiotropic costs (or heterozygous costs weaker than drive), novel centromeres may frequently sweep through populations unchecked, but precipitate later evolutionary change in CenH3 and other kinetochore proteins (e.g., after homogenization of all other chromosomes to a novel centromere sequence). This stepwise process may be rapid (and thus often undetectable) within populations but could produce species-level patterns of elevated divergence and be revealed as drive in interspecific hybrids. On the other hand, if recessive costs are a pleiotropic consequence of meiotic dysfunction when “strong” centromeres are homozygous, co-evolutionary dynamics such as those observed in polymorphic *M*. *guttatus* may be common. However, a balance between drive and costs that favors suppressor evolution should not deterministically cause joint centromere-CenH3 divergence at the species level, though it could lead to high levels of within species-variation and thus contribute to species differences eventually. Finally, our finding that the driving D is a large non-recombining haplotype including much more than centromeric repeats emphasizes the importance of structural and genic context for understanding centromeric drive and evolution. Rearrangements that suppress recombination around centromeres may be as important as the centromere repeats per se in the dynamics of chromosomal drive, both by altering centromere position or size and by creating opportunities for costly hitchhikers and linked enhancers. Thus, centromeric drive and kinetochore protein coevolution, and their consequences for individuals, populations, and species, may often be intertwined with the processes that shape the evolution of chromosome structure more broadly.

## Methods

### Genome sequencing, alignment, read mapping, and data filtering protocols

Whole genome re-sequence data (fastqs, Illumina reads) were obtained from the Sequence Read Archive (SRA) for 34 Iron Mountain (IM) inbred lines and four lines (AHQT, DUN, LMC24, and MAR3) from distant populations [24,35,36]. We generated new sequence data for two additional plants (one inbred line, one F_1_) derived from Iron Mountain. For the newly sequenced lines, DNA was extracted from fresh tissue using a modified CTAB-chloroform extraction protocol dx.doi.org/10.17504/protocols.io.bgv6jw9e. New genomic libraries were prepared following the Nextera tagmentation protocol and sequenced on the Illumina NextSeq platform (Ilumina NextSeq paired-end, 150 bp reads; Ilumina Inc., San Diego, USA), as described in [37]. All samples and their populations of origin, MDL11 haplotype call, and source are detailed in S2 Table. Note that IM712 was only included in linkage disequilibrium (LD) and depth of coverage analyses.

All sequences were quality- and adapter-trimmed with Trimmomatic version 0.35 [38] and aligned to the *M*. *guttatus* v2 reference genome (www.Phytozome.jgi.doe.gov) using bwa mem version 0.7.15 with default parameters [39]. Reads with mapping qualities less than 29 were filtered out with SAMtools v 1.3 [40] and duplicate reads were removed (Picard tools v 1.119; http://broadinstitute.github.io/picard). We used the Genome Analysis Toolkit (GATK) to re-align around indels and call variant sites with the Unified Genotyper tool, following GATK’s Best Practice recommendations [41,42]. Datasets were restricted to bi-allelic positions within genes using vcftools v0.1.12b [43], indels were removed, and sites covered by less than three reads per line were converted to missing data. For the highly inbred IM lines (mean H_OBS_ per individual = 0.041, SD = 0.01), we removed sites with any heterozygous genotype calls. For population genomic analyses, sites with genotype calls from at least 10 individuals were retained and genes with fewer than 150 retained sites were removed. Comparisons between IM and lines from distant populations (AHQT, DUN, LMC24, and MAR3) were restricted to sites retained in the IM population.

### Characterization of the MDL11 region

#### Scaffold re-ordering

For analyses of sequence variation on Linkage Group/Chromosome 11, we used a physical map based on the re-ordering of *M*. *guttatus* v1 scaffolds in a collinear (*D*^*-*^ x *D*^*-*^) IM767 x Point Reyes *M*. *guttatus* F_2_ mapping population [19,44]. In this mapping, v1 scaffolds were re-positioned, split, and inverted to optimize the genetic map, while retaining sequence and gene-annotation information for each v1 segment from the v2 assembly. In addition, we included the large (> 3 Mb) gene-poor v1 scaffold_10 in the MDL11 region (S1 Table), as it was placed there in v2 (and is clearly part of the *D* haplotype block in visual examination of Illumina-read alignments), but was lost from later genetic maps due to low genotyping quality in this repetitive region [19]. Mapped v1 scaffold sequences were extracted from the v2 reference genome and reordered into a new FASTA file based on their genetic coordinates. All gene sequences between contiguous genetically-mapped 100kb v1 segments were included in LD analyses (making them conservative; 1,188 genes), but divergent genes that were clearly not part of the MDL11 haplotype block (likely due to local mis-assembly) were excluded in remaining analyses unless specified (1,104 genes included, S4 Table).

#### Localization of *Cent728* satellite repeats and analyses of gene content

We used the Basic Local Alignment Search Tool (BLAST) [45] of the consensus nucleotide sequence of *Cent728* [14] to localize copies of the putative centromeric repeat on the re-ordered LG11. To survey for gene content differences (copy number variation; CNV) between *D* and *D*^*-*^ individuals across LG11, we used deviations in read depth following [46]. We allowed sites to have missing data and relaxed the read coverage per line criteria for these analyses. We used vcftools v0.1.12b [43] to obtain read depth for each exonic site (excluding indels, heterozygous sites, and sites with more than two alleles), standardized values by the individual’s chromosome-wide median for such sites, and calculated an average standardized read depth for each gene. Genes were excluded as likely misassembled or repetitive if *D* individuals had standardized coverage values < 0.5 or > 3, or if they were identified as chloroplast-nuclear transfers or nongenic mis-annotations in [46]. On LG11, 1,344 genes were retained. *D-*: *D* coverage ratios were used to categorize genes as likely deleted (0–0.25; red), duplicated (1.75–2.0; blue), or not likely duplicated or deleted (0.25–1.75; tan; Fig 1 bottom track).

#### Linkage disequilibrium, nucleotide diversity, and site frequency spectrum

To estimate linkage disequilibrium across LG11, we used vcftools version 1.12a [43] to calculate the squared correlation coefficient between genotypes (r^2^) for SNP pairs (N = 49,595 genic SNPs at IM). Average r^2^ across all polymorphic sites was then calculated for each gene pair (N = 1,475 genes). Second, we explored haplotype structure by calculating the proportion of SNPs per gene on LG11 that matched the IM62 reference for each sequenced line. For the haplotype structure analyses, we coded genes with fewer than seven polymorphic sites genotyped as missing data (N = 1,064 genes included). Average within-population nucleotide diversity (π) per gene, as well as *d*_*x*,*y*_ between *D* and *D*^*-*^ lines, was calculated in R using PopGenome [47], based on [22], and divided by the number of sites per gene. Calculations were performed separately for IM lines with *D* and *D*^*-*^ haplotypes, and values were averaged over MDL11 and flanking regions, respectively, in each. Genes inside MDL11 are listed in S4 Table; all other genes were considered to be in flanking regions. Confidence intervals were generated in the Hmisc package of R, version 4.1–1, by performing 1000 bootstrap re-samplings of the means without replacement [48].

#### Origin and age

To infer the demographic history of the MDL11 region, we applied pairwise sequentially Markovian coalescent (PSMC) analyses as implemented by [49]. Following [20], we created pseudo-diploids by combining haploid genomes from two inbred lines for estimation of pairwise coalescence and effective population size through time. To place *D* in context, we used two non-IM *D*^*-*^ lines with distinct evolutionary affinities: a coastal perennial individual derived from the Southern *M*. *guttatus* clade (DUN) and an annual representing the Northern *M*. *guttatus* clade (MAR), to which IM also belongs [20], as well as *D* (IM62) and *D*^*-*^ (IM767) IM lines. For this analysis, bams were first made as described in [37]. Pseudo-diploids were then created by making fasta files using the consensus sequence of each bam and merging the two consensus sequences using the seqtk toolset (https://github.com/lh3/seqtk)) with a quality threshold of 20. We performed 100 bootstrap replicates for each pairwise comparison. To perform the bootstraps, we applied the splitfa tool from the PSMC package to break the pseudo-diploids into non-overlapping chunks. The segmented genome then served as input for 100 separate PSMC analyses with the–b option. Coalescent analyses were performed separately for chromosomal locations within MDL11 and in flanking regions (S4 Table).

Because *D* is non-recombining with alternative alleles, we used mutation alone (rather than haplotype structure or a mix) to age it. First, to estimate the time since most recent common ancestor (t) for the *D* haplotype, we counted the number of segregating sites in 13 IM lines (IM62, IM115, IM116, IM138, IM1145, IM239, IM502, IM657, IM664, IM742, IM909, IM922, IM1054, excluding IM549 due to low coverage; S3 Table). We restricted this analysis to exonic sites where alignments are more reliable [24]. We excluded heterozygous sites and entire genes with >5 heterozygous exon sites, as these generally represent stacked copy number variants or other instances of incorrect alignment, which can also produce (apparently) homozygous SNPs.

To estimate the age of the swept *D* haplotype, we used both simple calculations [the Thomson estimator; 21]) and forward simulations using a range of mutation rates (0.2 x 10^−8^–1.5 x 10^−8^), following [20]. The Thomson estimator tends to underestimate time to the most recent common ancestor, as it does not include the initial spread of the focal haplotype to high frequency [21]; however, this is not a major concern given the short time to equilibrium frequency expected for driving *D* [12]. We also simulated mutation accumulation on a nonrecombining chromosome using the simulation software SLiM 2.6 The D haplotype contains a total of 256,867 nucleotide positions for which we have high-confidence genotype calls. We therefore simulated a population of nonrecombining chromosomes of length 256,867 bp that begins as a small founder population (n = 20 chromosomes) and grows exponentially by 10% per generation to a stable size of either 50,000 chromosomes (89 generations of growth) or 500,000 chromosomes (113 generations). We sampled 13 chromosomes per generation and counted the number of observed segregating sites in the sample. Simulations ended and the generation number was recorded when 9 segregating sites were observed. We performed simulations over a range of per-base mutation rates to correspond to population-scaled mutation rates (4Nμ) of 0.001 (1 x 10^−8^ per generation for 50,000 chromosomes, 1x10^-9^ for 500,000 chromosomes), which is well below observed π at Iron Mountain, to 0.01, which is similar to π at IM [24]. Simulation results are plotted in S2 Fig.

### Genetic mapping of loci underlying interspecific differences in vulnerability to *D* drive

#### Crossing design

To test for unlinked modifiers of LG11 *D* drive, we inter-crossed heterospecific *Dd* (SF *M*. *nasutus* x IM160) and *D*^*-*^*d* (SF *M*. *nasutus* x IM767) F_1_ hybrids to form an F_2_ mapping population. Because the SF x IM160 F_1_ (*Dd*) was used as the female parent, we expected these F_2_s to all be *D*d or *DD*^-^ (no *dd*, due to near-complete drive in the female *Dd* parent). Thus, we can examine the strength of heterospecific (*Dd*) and conspecific (*DD*^*-*^) drive in a segregating F_2_ background and map any major loci that modulate their expression. F_2_ individuals were grown in a greenhouse at the University of Montana under standard long-day growth conditions for *M*. *guttatus*, and DNA was extracted from leaf tissue for genotyping using our standard 96-well CTAB-chloroform protocol (dx.doi.org/10.17504/protocols.io.bgv6jw9e). We then categorized individuals as *DD*^*-*^ (conspecific drive heterozygote) or *Dd* (heterospecific drive heterozygote) using the diagnostic marker Lb5a [14].

#### Phenotyping

To characterize the strength of drive (the phenotype) in F_2_s, we hand self-pollinated 1–5 flowers per individual and collected the resultant selfed seeds. Some F_2_ hybrids set no seed, in part due to the segregation of known hybrid sterility factors [50] in this cross. For each selfed F_3_ seed family, we then planted 16 cells of a 96 well flat with 2 seeds each (or fewer if we did not have 32 viable seeds), and then thinned (and/or transplanted) to 16 per family. F_3_ plants were harvested as rosettes for DNA extraction and genotyping at diagnostic markers. Overall, we planted 250 progenies, and obtained 221 families (*Dd*; n = 101, *DD*^*-*^; n = 120) with at least 8 progeny successfully genotyped. For each progeny set, we estimated the strength of female meiotic drive (%D_fem_), assuming no distortion through male function (*Dd* expected > 0.98, *DD*^*-*^ expected = 0.58). This approach is not as precise as isolating female meiotic drive by hand-backcrossing F_2_s as dams (with prior emasculation in the bud) [13,14], but selfing was more tractable for the large number of small-flowered F_2_s involved.

#### Linkage and quantitative trait locus (QTL) mapping

We constructed a linkage map of the F_2_ population (n = 184 total genotyped; 91 included in linkage mapping set) using multiplex shotgun genotyping (MSG) to generate low-coverage genome sequence [51]. The GOOGA pipeline [19] was used to assign genotype probabilities to 100kb windows of the *M*. *guttatus* reference genome (v1 scaffolds; www.Phytozome.jgi.doe.gov) and order them into linkage groups corresponding to the 14 chromosomes of the *M*. *guttatus* and *M nasutus* genomes, as well as previous linkage maps of this interspecific cross [52,53]. As previously described [19], this approach corrects numerous ordering errors in the v2 chromosome-scale assembly of *M*. *guttatus*, while also allowing use of the v2 annotation through assignment of each 100kb v1 segment to its corresponding v2 segment. This process resulted in 1,836 physically and genetically mapped window-based markers.

For QTL mapping, we used the posterior probabilities generated by GOOGA [19] to make hard genotype calls for each 100kb genome window. Windows were assigned to one of the three fully informative genotypes (*M*. *guttatus* homozygote, *M*. *nasutus* homozygote, or heterozygote) if that genotype had a probability > 0.8. Windows that did not meet this criterion were called as missing. To verify that our genome-wide genotyping approach was effective, we tested for concordance between MDL11 windows and our D-diagnostic marker, excluding several individuals (likely contaminated during the MSG protocol and/or low coverage) where genotypes did not match. For QTL mapping of potential modifier loci, we restricted analyses to F_2_ individuals whose value of %D_fem_ was based on 12 or more F_3_ progeny, and who had <50% missing data (N = 130 total). We scanned for QTLs underlying %D_fem_ using the interval mapping function in WinQTLCart [54], with marker-based D genotype as a binary co-factor. We used a generous significance threshold of LOD = 2.0 (p < 0.05) for the initial scan.

To characterize the Chromosome/Linkage Group 14 (LG14) modifier QTL, we made an exon-primed marker (mCenH3A; S7 Table) that identified all three parental alleles of CenH3A –N from SF M. *nasutus*, G_160_ from IM160 and G_767_ from IM767 –based on length polymorphisms generated by intronic insertions and deletions. The two IM *M*. *guttatus* alleles were distinguished by a 1 basepair indel in the second intron. Because the crossing work pre-dated the sequencing of many inbred IM lines and the IM160 line was later lost, only an IM160 x IM767 F_1_ individual was available to sequence (S2 Table). However, it is apparent that the IM160 allele of CenH3A happened to be unusually divergent, with >22 Single Nucleotide Polymorphisms (SNPs) and/or indels in introns and UTRs, one synonymous SNP in Exon 1, and one nonsynonymous SNP in Exon 4 (part of the rapidly evolving N-terminal tail) relative to both the reference and IM767. We genotyped mCenH3A in 150 F_2_s with >12 progeny contributing to their %D_fem_ phenotype, and tested for effects of the four possible genotypes (NN, NG_160_, NG_767_, and G_160_ G_767_) using a two-way analysis of variance with mCENH3A genotype, MDL11 genotype, and their interaction as factors [55].

### Population genomics of CenH3A

Average pairwise nucleotide diversity (π) per site per gene and Tajima’s D per gene were calculated for genes on Chromosome 14 (N = 2703) in R using PopGenome [47], with the same parameters as for the analyses of Chromosome 11. CENH3A (Migut.N01557) resides in an 8-gene region of low diversity (Migut.N01552 –N01559; π < 0.005 in IM), which was also one of only 41 windows containing monophyletic-within-IM outliers in a previous study of selective sweeps at IM [24]. To further test whether such an extensive block of diversity reduction was extreme, we conducted permutations (N = 500) by calculating mean π for randomly chosen contiguous blocks of 8 genes along Chromosome 14. Confidence intervals were generated in the Hmisc package of R, version 4.0–2, by performing 1000 bootstrap re-samplings of the means without replacement [48].

To test whether diversity reduction around CenH3A at IM reflected low overall diversity, we also computed nucleotide diversity between samples from IM and distant populations, using the same approach as above. Calculations were performed sequentially between all IM lines and one other line (AQHT, DUN, MAR3, and LMC24), and confidence intervals generated as described above.

We visualized haplotype structure surrounding CenH3A using R version 3.5.0. Exonic SNPs on Chromosome 14 were phased using Beagle 4 [56] and the haplotypes surrounding CenH3A (scaffold positions 13,500,000–14,000,000) were converted to a matrix using a custom Python script (vcf2selscan.py). We included one haplotype per inbred line and plotted allelic states at each SNP relative to the IM1054 haplotype in R. Haplotypes were identified manually and their lengths are detailed in S6 Table.

To estimate the age of the CenH3A sweep from the length of surrounding haplotypes, we followed the approach of [57], using a range of local recombination rates (150kb-1000kb/cM based on genetic maps). Because we have a broad distribution of haplotype lengths, we calculated ages using the shortest shared core segment (24 kb), as well as the longest, shortest, mean and median haplotype lengths (S6 Table). The latter bookend the age of the shift in selection from 24 years (longest, least recombination) to 1598 years (shortest, most recombination). Because we do not currently have resolution to more finely estimate intra-population recombination rate, the key variable, we did not forward simulate this apparent sweep from standing variation.

### Confirmation of *D* vs. *D*^*-*^ gene content differences

Coverage differences between *D* and *D*^*-*^ lines at IM indicate that a 45-gene region is a) deleted in *D*^*-*^ relative to the (ancestral) *D* reference, b) inserted in *D* relative to ancestral *D*^-^ or c) so divergent that few or no reads from the *D*^*-*^ haplotype map to the *D* reference. The third alternative is unlikely, as exonic reads from across the species complex and beyond map well to exonic sequences in the IM62 reference [20,58]. To further rule out this possibility, we designed an exon-primed, intron-spanning, length polymorphic PCR marker in the RCY1 homolog Migut.K01228/Migut.K01229 (mK1229; S7 Table). This marker also amplifies a fragment from a second RCY1 gene on LG10 (Migut.J00575), which acts as a positive control for amplification of the sample. We genotyped 120 wild-derived greenhouse-grown IM outbred plants using touchdown PCR amplification of fluorescently-tagged fragments sized with capillary electrophoresis on an ABI 3130 Genetic Analyzer [13]. A 173bp fragment from Migut.K01229 segregated as a presence/absence polymorphism in perfect association with our standard MDL11 diagnostic marker for the IM population (Lb5a), while the 180bp band from Migut.J00575 was present in all individuals. This pattern (along with the low coverage shown in Fig 1) suggests that the *D*^*-*^ plants do indeed lack sequence in this region.

## Supporting information

S1 Fig The driving *D* haplotype of MDL11 is an extended region of sequence identity to the reference *M*. *guttatus* genome. Each colored block represents a re-ordered gene on Chromosome/Linkage Group 11, colored to indicate the proportion of SNPs that match the reference IM62 (*D*) line (N = 1,064; genes with insufficient data are coded in white). Vertical lines bound the first and last gene of the MDL11 region (Migut.K01047 to Migut.K00885; S4 Table). Horizontal tracks represent the haplotypes of 34 inbred lines isolated from the IM population, with *D* lines sorted to the top (from top to bottom: IM62, IM115, IM239, IM549, IM657, IM742, IM502, IM138, IM1054, IM922, IM909, IM664, IM116, IM1145, IM835, IM767, IM693, IM624, IM479, IM109, IM785, IM777, IM709, IM667, IM275, IM266, IM238, IM179, IM170, IM1192, IM1152, IM359, IM106, IM412).
(TIF)

S2 Fig Simulated and analytical results point to a recent origin of the *D* haplotype. Forward simulations were performed using SLiM 2 (described in Materials and Methods) over a range of mutation rates and with equilibrium census population sizes (N_C_) of 25,000 (50,000 *D* chromosomes, top panel) and 250,000 diploids (bottom panel). Mutation rates are scaled by N_C_ in the figure and correspond to ranges of 1x10^-8^-1x10^-7^ (top) and 1x10^-9^-1x10^-8^ (bottom). Grayscale density reflects the proportion of simulations yielding a T_MRCA_ (time to most recent common ancestor) of the *D* haplotype within each bin. Gold shading represents the range of *D* haplotype ages calculated using the Thomson estimator (Thomson et al. 2000).
(TIF)

S3 Fig The pairwise sequentially Markovian coalescent (PSMC) method suggests that *D* and *D*^*-*^ alleles share a similar demographic history across LG11. PSMC inference of population size through time for pairwise haploid genome comparisons in the A) MDL11 region and B) the flanking regions of LG11. In B), the inset is a zoomed-out view of PSMC simulations. Haploid genomes of two inbred lines were used to create pseudo-diploids to use for estimating coalescence. Color codes are as follows: red = *D* line (IM62) x Southern-clade *M*. *guttatus* line (DUN); green = *D*^*-*^ (IM767) line x southern *M*. *guttatus* (DUN); blue = *D* (IM62) line x *D*^*-*^ (IM767) line; purple = *D* (IM62) line x northern *M*. *guttatus* (MAR); teal = *D*^*-*^ (IM767) line x northern *M*. *guttatus* (MAR). Thick lines represent the point inference and thin lines represent bootstrap replicates (N = 100).
(TIF)

S4 Fig Low nucleotide diversity and a skewed site frequency spectrum show a recent CenH3A selective sweep in the IM population (N = 34 lines). A) Histogram of permuted means calculated by averaging π per site per gene from blocks of 8 consecutive genes along LG14. Permutations were performed 500 times. B) Histogram of permuted means calculated by averaging Tajima’s D per gene from blocks of 8 consecutive genes along LG14, which contains CenH3A. Permutations were performed 500 times.
(TIF)

S1 Table The scaffold of LG11 are re-ordered based on a collinear *D*^*-*^ x *D*^*-*^ map (Flagel et al. [19]). For each *M*. *guttatu*s v1 scaffold on LG11, the table shows assignment of its genes to *D* (1), *D*^*-*^ (0) or both (REC), its v2 assembly position and genes, its orientation in the new map order, whether or not the v1 scaffold needed to be split, and its
(DOCX)

S2 Table The lines (inbred except for IM160xIM767 F_1_) used in this study are listed, with their population of origin, MDL11 haplotype, source and Sequence Read Archive accession number. (XLSX)

S3 Table Nine exonic mutations (bolded) in 13 IM lines (columns) were used for estimating the time since the *D* selective sweep. The ANC column contains the inferred sequence of the common ancestor of all sampled *D*
(DOCX)

S4 Table Genes in the MDL11 region are listed, with *Mimulus guttatus* v2 assembly/annotation number, order in the reordered LG11 map, *Arabidopsis thaliana* best-hit homologue name, and gene descriptions from Phytozome 12.1. (XLSX)

S5 Table Inter- and intra-population nucleotide diversity levels at CenH3A^a^ are compared to other regions on LG14. (DOCX)

S6 Table Seven distinct, long-range haplotypes carried by more than one individual define the CenH3A region of LG14. Start and end coordinates on LG14, size in base pairs, IM individuals with the haplotype, and the number of individuals are given for haplotypes 1–7, as well as the identities of four singleton lines with unique haplotypes.
(DOCX)

S7 Table Marker names, genes, primer sequences, and product sizes are listed for genetic markers used in this study. The MDL11 marker lb5a has multiple non-reference (*D*) alleles, mK1229/J575 is a presence/absence polymorphism, and the lengths of the *N*, *G*_*767*_, and*G*_*160*_ allele at mCenH3A are 294, 285, and 287 bases, respectively.
(DOCX)
