# Supplementary figures and images for: Selfish chromosomal drive shapes recent centromeric histone evolution in monkeyflowers
Source: PLoS Genet. 2021 Apr 22;17(4):e1009418. doi: 10.1371/journal.pgen.1009418 (PMC8061799; doi:10.1371/journal.pgen.1009418)

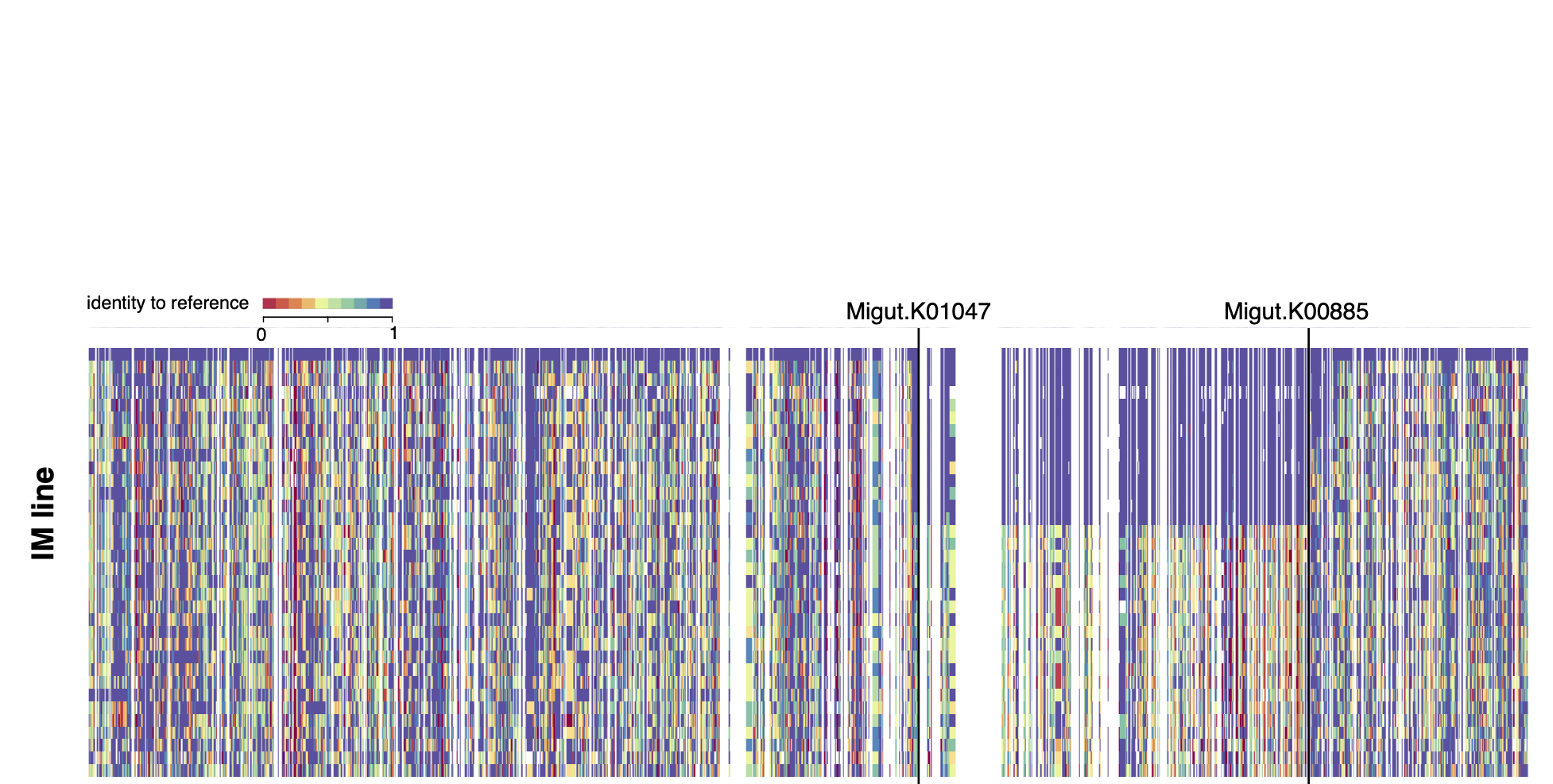

Supplement: S1 Fig — Each colored block represents a re-ordered gene on Chromosome/Linkage Group 11, colored to indicate the proportion of SNPs that match the reference IM62 (D) line (N = 1,064; genes with insufficient data are coded in white). Vertical lines bound the first and last gene of the MDL11 region (Migut.K01047 to Migut.K00885; S4 Table). Horizontal tracks represent the haplotypes of 34 inbred lines isolated from the IM population, with D lines sorted to the top (from top to bottom: IM62, IM115, IM239, IM549, IM657, IM742, IM502, IM138, IM1054, IM922, IM909, IM664, IM116, IM1145, IM835, IM767, IM693, IM624, IM479, IM109, IM785, IM777, IM709, IM667, IM275, IM266, IM238, IM179, IM170, IM1192, IM1152, IM359, IM106, IM412). (TIF) [file pgen.1009418.s001.tif]

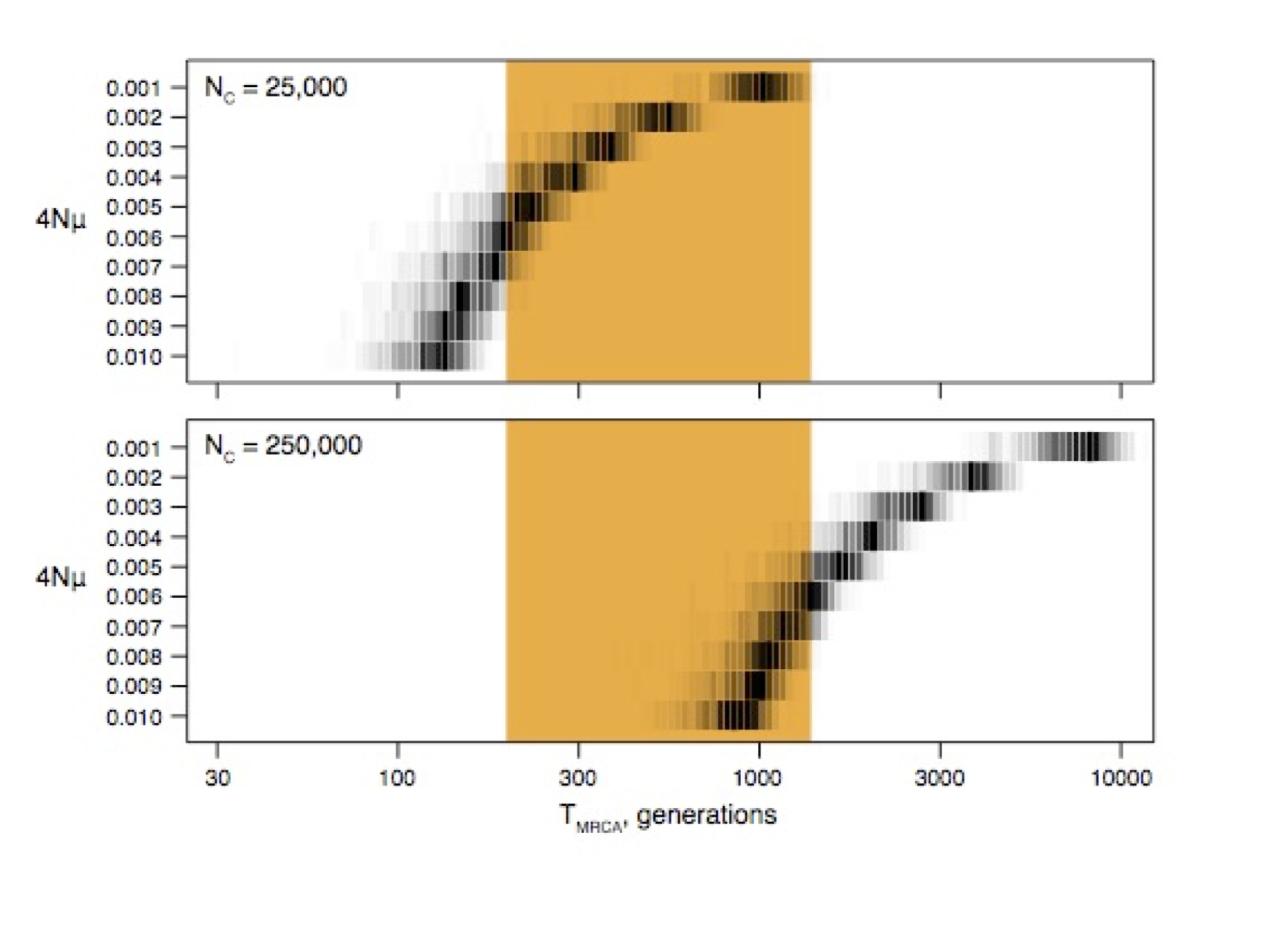

Supplement: S2 Fig — Forward simulations were performed using SLiM 2 (described in Materials and Methods) over a range of mutation rates and with equilibrium census population sizes (NC) of 25,000 (50,000 D chromosomes, top panel) and 250,000 diploids (bottom panel). Mutation rates are scaled by NC in the figure and correspond to ranges of 1x10^-8^-1x10-7 (top) and 1x10^-9^-1x10-8 (bottom). Grayscale density reflects the proportion of simulations yielding a TMRCA (time to most recent common ancestor) of the D haplotype within each bin. Gold shading represents the range of D haplotype ages calculated using the Thomson estimator (Thomson et al. 2000). (TIF) [file pgen.1009418.s002.tif]

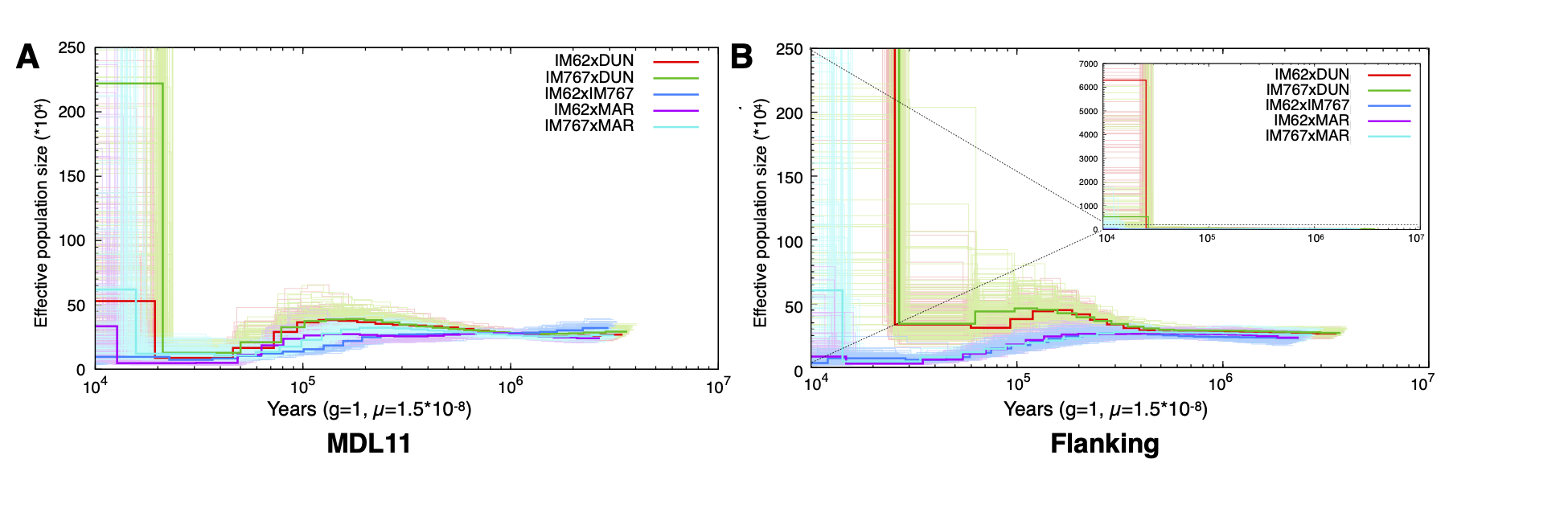

Supplement: S3 Fig — PSMC inference of population size through time for pairwise haploid genome comparisons in the A) MDL11 region and B) the flanking regions of LG11. In B), the inset is a zoomed-out view of PSMC simulations. Haploid genomes of two inbred lines were used to create pseudo-diploids to use for estimating coalescence. Color codes are as follows: red = D line (IM62) x Southern-clade M. guttatus line (DUN); green = D- (IM767) line x southern M. guttatus (DUN); blue = D (IM62) line x D- (IM767) line; purple = D (IM62) line x northern M. guttatus (MAR); teal = D- (IM767) line x northern M. guttatus (MAR). Thick lines represent the point inference and thin lines represent bootstrap replicates (N = 100). (TIF) [file pgen.1009418.s003.tif]

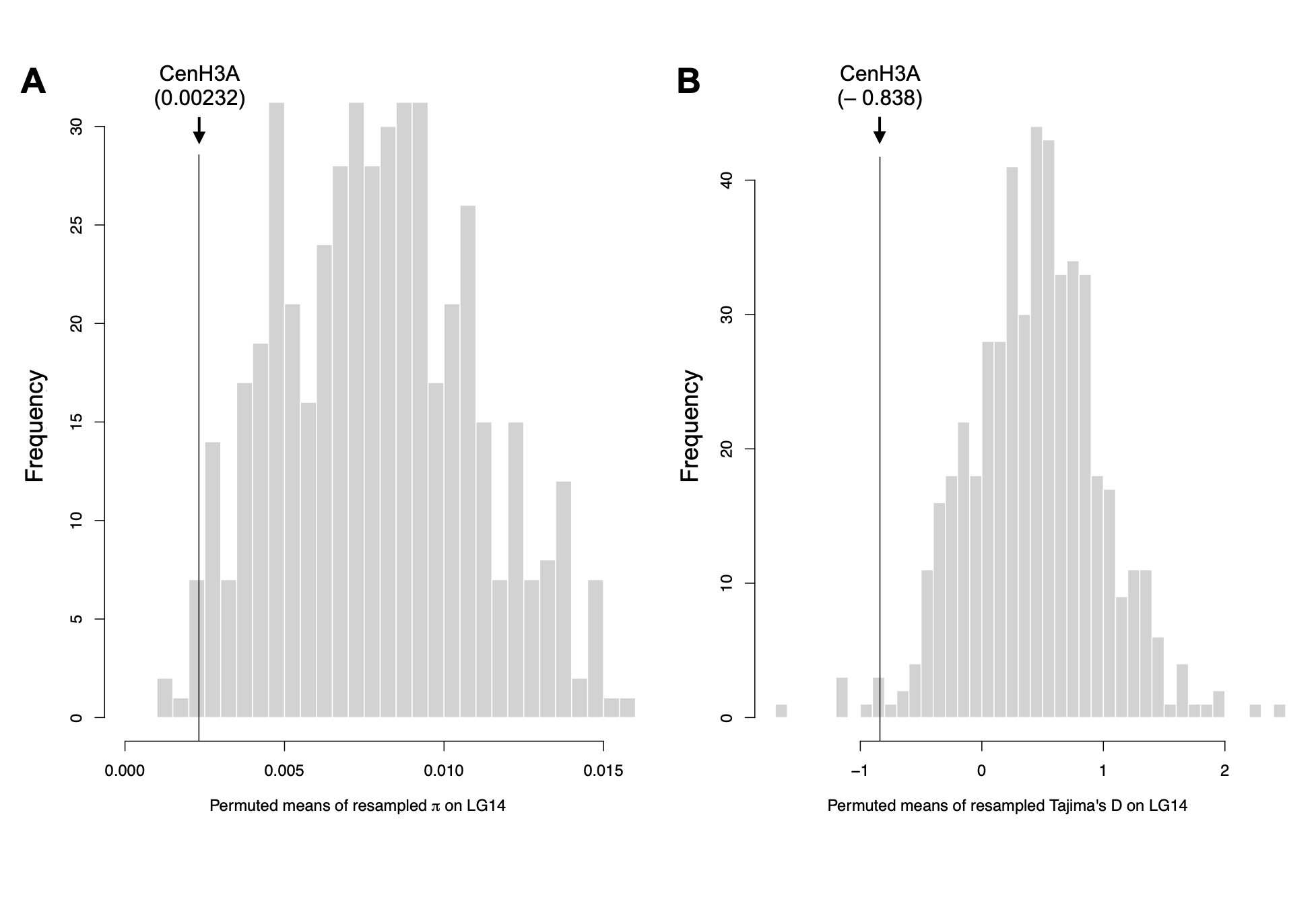

Supplement: S4 Fig — A) Histogram of permuted means calculated by averaging π per site per gene from blocks of 8 consecutive genes along LG14. Permutations were performed 500 times. B) Histogram of permuted means calculated by averaging Tajima’s D per gene from blocks of 8 consecutive genes along LG14, which contains CenH3A. Permutations were performed 500 times. (TIF) [file pgen.1009418.s004.tif]
