# Supplementary material for: Selfish chromosomal drive shapes recent centromeric histone evolution in monkeyflowers
Source: PLoS Genet. 2021 Apr 22;17(4):e1009418. doi: 10.1371/journal.pgen.1009418 (PMC8061799; doi:10.1371/journal.pgen.1009418)
Supplement: S1 Table — For each M. guttatus v1 scaffold on LG11, the table shows assignment of its genes to D (1), D- (0) or both (REC), its v2 assembly position and genes, its orientation in the new map order, whether or not the v1 scaffold needed to be split, and its (DOCX) [file pgen.1009418.s005.docx]

| v1  scaffold | MDL11? | | v2  start | | v2  end | | Gene  start | | Gene  end | | v2  orient | v1  orient | | v1 split | v1  length | |  |  |  |
| --- | --- | --- | --- | --- | --- | --- | --- | --- | --- | --- | --- | --- | --- | --- | --- | --- | --- | --- | --- |
| 75 | 0 | | 0 | | 1159689 | | K00001 | | K00254 | | F | F | | N | 1159689 | |  |  |  |
| 228 | 0 | | 1169690 | | 1556838 | | K00255 | | K00334 | | F | F | | N | 387148 | |  |  |  |
| 273 | 0 | | 1566839 | | 1750548 | | K00335 | | K00367 | | F | F | | Y | 183709 | |  |  |  |
| 213 | 0 | | 1760548 | | 2177483 | | K00368 | | K00445 | | F | F | | N | 416935 | |  |  |  |
| 63 | 0 | | 2430218 | | 2187484 | | K00485 | | K00446 | | R | R | | Y | 242734 | |  |  |  |
| 257 | 0 | | 2605914 | | 2440218 | | K00513 | | K00486 | | R | F | | Y | 165696 | |  |  |  |
| 30 | 0 | | 2615914 | | 4575289 | | K00514 | | K00667 | | F | R | | N | 1959375 | |  |  |  |
| 48 | 0 | | 5742497 | | 4831822 | | K00736 | | K00668 | | R | F | | Y | 910675 | |  |  |  |
| 243 | 0 | | 13523272 | | 13853907 | | K00948 | | K00969 | | F | F | | N | 330635 | |  |  |  |
| 415 | 0 | | 14223853 | | 14300664 | | K00984 | | K00987 | | F | R | | N | 76811 | |  |  |  |
| 182 | 0 | | 14427980 | | 14963817 | | K00994 | | K01026 | | F | F | | N | 535837 | |  |  |  |
| 185 | 0 | | 15549682 | | 15047665 | | K01042 | | K01032 | | R | F | | N | 502017 | |  |  |  |
| 39 | REC | | 16402937 | | 15559683 | | K01078 | | K01043 | | R | F | | Y | 843254 | |  |  |  |
| 47 | 1 | | 18948653 | | 17891937 | | K01153 | | K01123 | | R | R | | Y | 1056716 | |  |  |  |
| 6 | 1 | | 22410203 | | 21063652 | | K01260 | | K01208 | | R | F | | Y | 1346551 | |  |  |  |
| 145 | 1 | | 11601986 | | 12393390 | | K00944 | | K00944 | | F | F | | N | 791404 | |  |  |  |
| 49 | 1 | | 19506753 | | 21053901 | | K01154 | | K01207 | | F | R | | N | 1547148 | |  |  |  |
| 167 | 1 | | 6237216 | | 5673894 | | K00751 | | K00737 | | R | R | | N | 563322 | |  |  |  |
| 10 | 1 | | 6247217 | | 9226930 | | K00752 | | K00759 | | F | R | | N | 2979713 | |  |  |  |
| 239 | 1 | | 13863908 | | 14213852 | | K00970 | | K00983 | | F | F | | N | 349944 | |  |  |  |
| 131 | 1 | | 16412937 | | 17176752 | | K01079 | | K01105 | | F | R | | N | 763815 | |  |  |  |
| 221 | 1 | | 17397001 | | 17186753 | | K01115 | | K01106 | | R | R | | Y | 210248 | |  |  |  |
| 100 | 1 | | 11591985 | | 10601358 | | K00943 | | K00886 | | R | F | | N | 990627 | |  |  |  |
| 162 | 1 | | 9900780 | | 9236931 | | K00809 | | K00760 | | R | F | | N | 663849 | |  |  |  |
| 161 | 1 | | 10006245 | | 10591357 | | K00816 | | K00885 | | F | R | | N | 585112 | |  |  |  |
| 22 | REC | | 22846476 | | 24977148 | | K01261 | | K01499 | | F | F | | Y | 2130672 | |  |  |  |
|  | |  | |  | |  | |  | |  | | |  | | |  | |  |  |
|  | |  | |  | |  | |  | |  | | |  | | |  | |  |  |
