# Supplementary material for: Selfish chromosomal drive shapes recent centromeric histone evolution in monkeyflowers
Source: PLoS Genet. 2021 Apr 22;17(4):e1009418. doi: 10.1371/journal.pgen.1009418 (PMC8061799; doi:10.1371/journal.pgen.1009418)
Supplement: S3 Table — The ANC column contains the inferred sequence of the common ancestor of all sampled D (DOCX) [file pgen.1009418.s007.docx]

| v2 position | ANC | 1054 | 115 | 116 | 138 | 664 | 239 | 502 | 657 | 742 | 909 | 922 | 1145 | 62 |
| --- | --- | --- | --- | --- | --- | --- | --- | --- | --- | --- | --- | --- | --- | --- |
| 9413102 | G | G | G | G | G | G | G | G | G | G | G | G | **T** | G |
| 9420494 | T | T | T | T | T | T | T | T | T | T | T | T | **C** | T |
| 9664721 | G | G | G | G | G | G | G | G | G | G | G | **A** | G | G |
| 10228601 | A | A | A | A | **T** | A | A | A | A | A | A | A | A | A |
| 10739451 | A | A | A | A | A | A | **C** |  | A | A | A | A | A | A |
| 16858792 | T | T | T | T | T | T | T | T | T | T | T | **A** | T | T |
| 19962104 | G | G | G | G | G | G | G | G | G | G | G | G | **A** | G |
| 20024342 | C | C | C | C | C | C | C | C | C | **T** | C | C | C | C |
| 21424794 | A | **G** |  | A | A | A | A | A | A | A | A | A | A | **G** |
