# Supplementary material for: Selfish chromosomal drive shapes recent centromeric histone evolution in monkeyflowers
Source: PLoS Genet. 2021 Apr 22;17(4):e1009418. doi: 10.1371/journal.pgen.1009418 (PMC8061799; doi:10.1371/journal.pgen.1009418)
Supplement: S5 Table — (DOCX) [file pgen.1009418.s009.docx]

| **Comparison^b^** | **Mean 𝝅^c^** | **Mean of permuted means^d^** | **Range of permuted means** | **Standard deviation of permuted means** | ***P*^e^** |
| --- | --- | --- | --- | --- | --- |
| IM-IM | 0.00232 | 0.00796 | (0.00109 - 0.01920) | 0.00014 | **0.016** |
| IM-AHQT | 0.00413 | 0.00909 | (0.00172 - 0.02229) | 0.00017 | 0.086 |
| IM-DUN | 0.00488 | 0.00708 | (0.00088 - 0.01407) | 0.00011 | 0.204 |
| IM-LMC24 | 0.00630 | 0.00899 | (0.00070 - 0.01945) | 0.00014 | 0.224 |
| IM-MAR3 | 0.00461 | 0.00920 | (0.00098 - 0.02331) | 0.00018 | 0.116 |

| ^a^Diversity values computed for 8-gene block surrounding CenH3A |
| --- |
| ^b^ 34 IM lines and a line from a distant population (Table S1) |

^c^ Nei's diversity per gene per site (Nei 1979)

^d^500 permutations were performed by averaging π per site per gene from blocks of 8 consecutive genes along LG14

^e^P values generated by comparing estimated means to permuted means
