# Supplementary material for: Selfish chromosomal drive shapes recent centromeric histone evolution in monkeyflowers
Source: PLoS Genet. 2021 Apr 22;17(4):e1009418. doi: 10.1371/journal.pgen.1009418 (PMC8061799; doi:10.1371/journal.pgen.1009418)
Supplement: S6 Table — Start and end coordinates on LG14, size in base pairs, IM individuals with the haplotype, and the number of individuals are given for haplotypes 1–7, as well as the identities of four singleton lines with unique haplotypes. (DOCX) [file pgen.1009418.s010.docx]

| **Haplotype** | **Start position** | **End position** | **Size (bp)** | **Individuals** | **N** |
| --- | --- | --- | --- | --- | --- |
| 1 | 13836336 | 13581975 | 254361 | IM1054, IM239, IM275, IM170, IM767, IM835, IM742 | 7 |
| 2 | 13105111 | 13725184 | 620073 | IM667, IM664, IM1192 | 3 |
| 3 | 13770309 | 13551509 | 218800 | IM549, IM359 | 2 |
| 4 | 13651251 | 13713827 | 62576 | IM624, IM62, IM179, IM693, IM777, IM479, IM709 | 7 |
| 5 | 13581975 | 13720468 | 138493 | IM109, IM922, IM238, IM115, IM909, IM657 | 6 |
| 6 | 13555923 | 13720169 | 164246 | IM1145, IM785, IM116 | 3 |
| 7 | 13506401 | 13902746 | 396345 | IM412, IM138 | 2 |
| singletons | N/A | N/A | N/A | IM266, IM502, IM1152, IM106 | 1 each |
