# Supplementary material for: Selfish chromosomal drive shapes recent centromeric histone evolution in monkeyflowers
Source: PLoS Genet. 2021 Apr 22;17(4):e1009418. doi: 10.1371/journal.pgen.1009418 (PMC8061799; doi:10.1371/journal.pgen.1009418)
Supplement: S7 Table — The MDL11 marker lb5a has multiple non-reference (D) alleles, mK1229/J575 is a presence/absence polymorphism, and the lengths of the N, G767, andG160 allele at mCenH3A are 294, 285, and 287 bases, respectively. (DOCX) [file pgen.1009418.s011.docx]

| Marker | Gene | Forward Primer | Reverse Primer | Ref allele size (in bases) |
| --- | --- | --- | --- | --- |
| lb5a | Migut.K00858 | CGGAGAATATATCGTGGTGG | ACTGCACCTCTCAATCTTGG | 276 |
| mK1229/J575 | Migut.K01229/  Migut.J00575 | TGTGGATCTAAAGGGAGATTTGA | TCATTTGCAAGATTCCATGC | 173 /180 |
| mCenH3A | Migut.N01557 | AAGAAATCCTCCGGTGAGAA | AACATGGTGTAGCAGTTGTGC | 274 |
